# Integrated Multimodal Analyses of DNA Damage Response and Immune Markers as Predictors of Response in Metastatic Triple-Negative Breast Cancer in the TNT Trial (NCT00532727)

**DOI:** 10.1158/1078-0432.CCR-23-0370

**Published:** 2023-08-14

**Authors:** Holly Tovey, Orsolya Sipos, Joel S. Parker, Katherine A. Hoadley, Jelmar Quist, Sarah Kernaghan, Lucy Kilburn, Roberto Salgado, Sherene Loi, Richard D. Kennedy, Ioannis Roxanis, Patrycja Gazinska, Sarah E. Pinder, Judith Bliss, Charles M. Perou, Syed Haider, Anita Grigoriadis, Andrew Tutt, Maggie Chon U. Cheang

**Affiliations:** 1 Clinical Trials and Statistics Unit, The Institute of Cancer Research, London, United Kingdom.; 2 Breast Cancer Now Toby Robinsons Research Centre, The Institute of Cancer Research, London, United Kingdom.; 3 Department of Genetics, Lineberger Comprehensive Cancer Center, University of North Carolina at Chapel Hill, Chapel Hill, North Carolina.; 4 The Breast Cancer Now Unit, King's College London Faculty of Life Sciences and Medicine, London, United Kingdom.; 5 School of Cancer and Pharmaceutical Sciences, King's College London Faculty of Life Sciences and Medicine, London, United Kingdom.; 6 Department of Pathology, GZA-ZNA Hospitals, Antwerp, Belgium.; 7 Peter MacCallum Cancer Centre, University of Melbourne, Melbourne, Victoria, Australia.; 8 ALMAC Diagnostic Services, Northern Ireland, United Kingdom.; 9 Biobank Research Group, Lukasiewicz Research Network – PORT Polish Center for Technology Development, Wroclaw, Poland.

## Abstract

**Purpose:**

The TNT trial (NCT00532727) showed no evidence of carboplatin superiority over docetaxel in metastatic triple-negative breast cancer (mTNBC), but carboplatin benefit was observed in the germline *BRCA1/2* mutation subgroup. Broader response-predictive biomarkers are needed. We explored the predictive ability of DNA damage response (DDR) and immune markers.

**Experimental Design:**

Tumor-infiltrating lymphocytes were evaluated for 222 of 376 patients. Primary tumors (PT) from 186 TNT participants (13 matched recurrences) were profiled using total RNA sequencing. Four transcriptional DDR-related and 25 immune-related signatures were evaluated. We assessed their association with objective response rate (ORR) and progression-free survival (PFS). Conditional inference forest clustering was applied to integrate multimodal data. The biology of subgroups was characterized by 693 gene expression modules and other markers.

**Results:**

Transcriptional DDR-related biomarkers were not predictive of ORR to either treatment overall. Changes from PT to recurrence were demonstrated; in chemotherapy-naïve patients, transcriptional DDR markers separated carboplatin responders from nonresponders (*P* values = 0.017; 0.046). High immune infiltration was associated with docetaxel ORR (interaction *P* values < 0.05). Six subgroups were identified; the immune-enriched cluster had preferential docetaxel response [62.5% (D) vs. 29.4% (C); *P* = 0.016]. The immune-depleted cluster had preferential carboplatin response [8.0% (D) vs. 40.0% (C); *P* = 0.011]. DDR-related subgroups were too small to assess ORR.

**Conclusions:**

High immune features predict docetaxel response, and high DDR signature scores predict carboplatin response in treatment-naïve mTNBC. Integrating multimodal DDR and immune-related markers identifies subgroups with differential treatment sensitivity. Treatment options for patients with immune-low and DDR-proficient tumors remains an outstanding need. Caution is needed using PT-derived transcriptional signatures to direct treatment in mTNBC, particularly DDR-related markers following prior chemotherapy.

**Translational Relevance**

Therapies targeting aberrant DNA damage response (DDR) have improved outcomes in metastatic triple-negative breast cancer (mTNBC) with a *BRCA1/*2 or *PALB2* mutation, but better predictive biomarkers are needed. We explored the predictive ability of primary tumor (PT)–derived transcriptional biomarkers of DDR biology and tumor immune microenvironment (TIME) following carboplatin or docetaxel in mTNBC. Data and samples were from the TNT Trial, which randomized women with mTNBC or a g*BRCA1/2* mutation between docetaxel and carboplatin. TIME biomarkers predicted preferential response to docetaxel. DDR signature scores were higher in recurrence than PT. DDR signatures predicted carboplatin response only in chemotherapy-naïve patients. Integrative analyses combining TILs, transcriptional, and somatic genetic features identified subgroups with differential treatment sensitivity. Results highlight the potential and complexity of studying biomarkers in PT for treatment selection in the advanced setting. PT-derived biomarker effects should be interpreted cautiously given the selective nature of this cohort who all developed metastatic disease.

## Introduction

We previously reported results of the TNT trial that showed patients with a *BRCA1/2* mutation and locally advanced/metastatic triple-negative breast cancer (TNBC) had improved response to carboplatin compared with docetaxel, with no significant selective benefit for carboplatin observed in the unselected population or those subcategorized by *BRCA1* methylation, HRD status, or basal-like subtype (1). Similar objective response rates (ORR) and progression-free survival with the use of PARP inhibitors to those observed for carboplatin in TNT in the metastatic setting have been shown for patients with germline *BRCA1/2* mutations (2–4). GeparSIXTO and BRIGHTness showed a benefit of neoadjuvant platinum therapy in patients with TNBC; however, the benefits were not restricted to *gBRCA1/2* mutation carriers (5–7). Analyses of effects of germline *BRCA1/2* mutation and/or presence of tumor-based HRD mutational signatures are complex showing a lack of prediction of carboplatin benefit, likely confounded by the frequency of epigenetic and genetic causes of HRD operating in a treatment-naïve context (8) as discussed in our previous manuscript (1). The PARP inhibitor olaparib has been shown to improve distant and overall survival in patients with germline *BRCA1/2* mutations in the adjuvant setting (9, 10). Given the relatively low frequency of *BRCA1/2* mutations in breast cancer, there is a need to identify additional predictive biomarkers in TNBC patients that relate specifically to response and survival with these mechanistically distinct forms of chemotherapy.

Several putative genomic markers of homologous recombination (HR) DNA repair deficiency (HRD), sometimes termed “BRCAness”, have been developed to act as patient selection biomarkers for treatment with therapies targeting aberrant DNA damage response. Both HRDetect, a whole-genome sequencing–based signature (11), and the HRD score, combining information about HRD-loss of heterozygosity (LOH), HRD-telomeric allelic imbalance, and HRD-large-scale state transitions (12), were shown to be associated with *BRCA1/2* mutation status. HRD score was prognostic in patients with TNBC treated with standard neoadjuvant chemotherapy (13) and those treated with platinum-containing regimes (14). Similarly, in ovarian cancer, the Foundation medicine assay, based on LOH, was explored in the ARIEL studies (15, 16). Within *BRCA*1/2 wild-type patients, while both groups benefitted from the addition of rucaparib, a benefit was greater in the LOH-high cohort compared with LOH-low (15, 16). Although associated with BRCAness, the HRD score was not associated with single agent platinum response in advanced TNBC in the TNT trial (1). However, we subsequently showed that intermediate telomeric NtAI (17), intermediate allelic imbalanced CNA (AiCNA) or those without high-level amplifications (HLAMP) had a moderate but significant interaction with platinum-specific objective response (18).

Genomic scars in the somatic tumor genome report historic and persistently active DDR activities or deficiencies, and therefore, might be insensitive as predictors in the presence of reversion of DDR function such *BRCA1* or *BRCA2* “reversion mutations” or loss of methylation of *BRCA1 (**19*) and may not be optimum for directing treatment options. Transcriptional signatures, however, reflect active DDR deficiencies, at least at the time of biopsy. Several promising signatures are proposed (20–23) but have not yet been adequately assessed in a controlled clinical trial of single-agent platinum treatment with an appropriate control group.

Beyond markers of BRCAness, breast tumors with DDR deficiencies have been associated with increased immune activity, with important links identified between DDR activity and the tumor microenvironment (24, 25). However, other studies have demonstrated the association between tumor-infiltrating lymphocytes (TIL) and BRCAness does not hold within TNBC tumors (26), so the exact relationship remains unclear. Despite this, TILs are prognostic in TNBC (27, 28) and predictive of response to immunotherapies (29, 30). It is not yet clear whether a possible association with DDR deficiencies may lead to the specific benefit of DDR-targeted treatments in tumors with high immune infiltration. There is some evidence that taxanes may be beneficial in these tumors by inducing immunogenic cell death (31).

Here, we aim to explore transcriptional markers of DDR deficiency and immune profiles in the available archival primary tumour samples in patients of the TNT trial and to perform exploratory analyses to test the hypothesis that tumors with high expression of DDR markers are preferentially sensitive to carboplatin over docetaxel while tumors with high expression of immune markers are sensitive to either treatment when treated in the locally advanced or metastatic setting.

## Materials and Methods

### Patients and samples

We analyzed total RNA sequencing and TILs on all available primary tumors (PT) and matched recurrence samples from TNT (1). Details of the main TNT trial, including inclusion and exclusion criteria, demographics, treatment regimens, and randomization are available and reported (1). In brief, TNT recruited 376 women with breast cancer aged between 26 and 81. Key inclusion criteria were a histologically confirmed diagnosis of ER-, PgR-, and HER2- primary breast cancer or the presence of a germline *BRCA1/2* mutation (regardless of ER/PgR/HER2 status) and no prior use of platinum-based chemotherapy. Patients entered the study at the point of first metastatic relapse or diagnosis of advanced inoperable local disease. However, patients could be recruited at their second metastatic relapse following progression on a non-taxane, anthracycline-based treatment if they had not received anthracyclines in the adjuvant setting. Patients were randomized to receive docetaxel or carboplatin, treatment allocation was not blinded.

**Figure 1.**
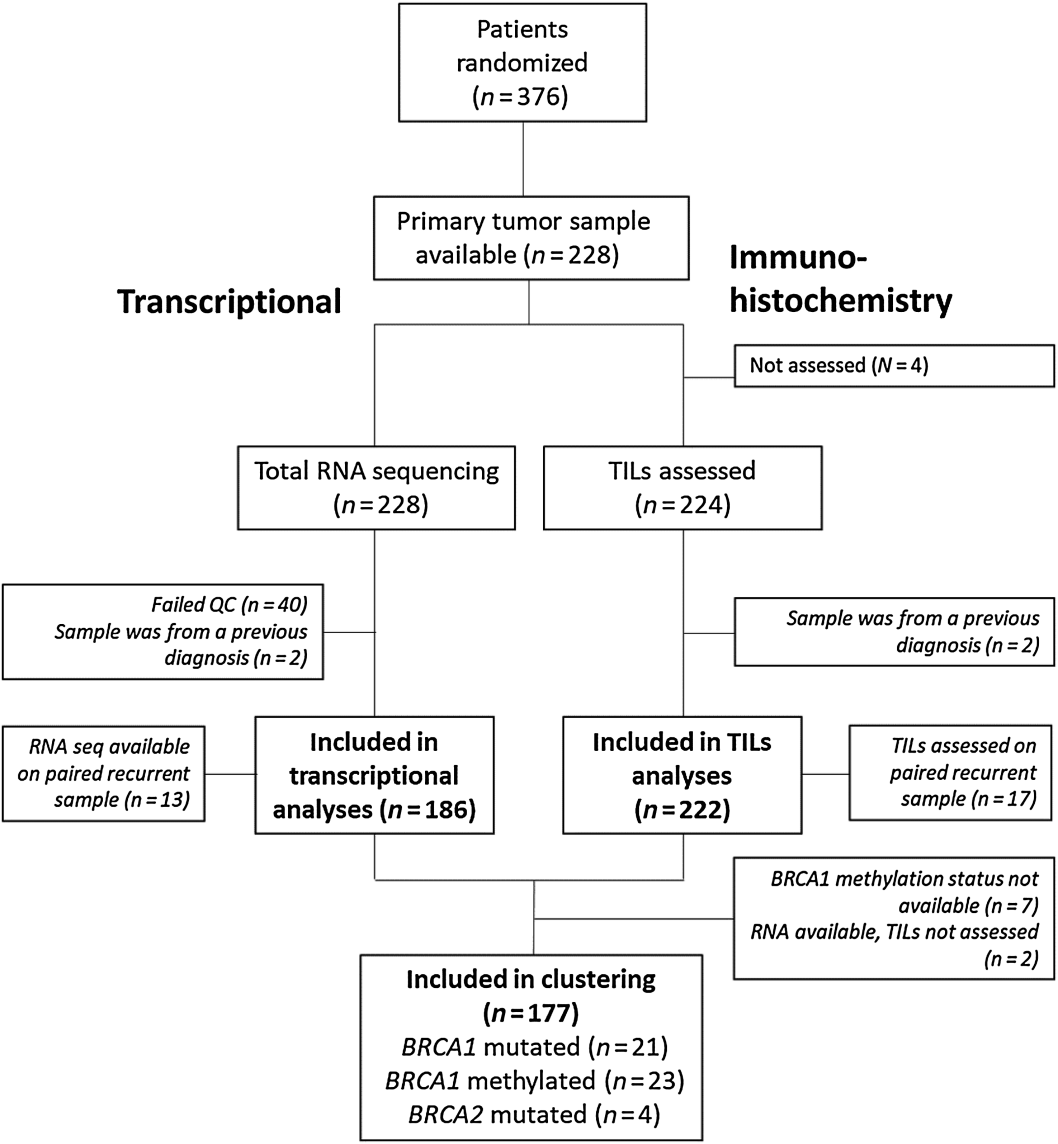
CONSORT diagram showing the number of primary tumor samples included in analyses and reasons for exclusions.

RNA sequencing data are available for PT from 186 patients and TILs from 222 patients (Fig. 1). RNA sequencing was available for matched recurrence samples for 13 patients and TILs for 17. RNA was extracted from tissue samples following standard methods for formalin-fixed paraffin-embedded (FFPE) samples and total RNA sequencing was carried out at the University of North Carolina at Chapel Hill using Illumina HiSeq2000 machine; methods have been reported previously (1). TILs were assessed using the digital image of a single H&E-stained slide in ndpi format obtained on the Nanozoomer HT scanner (Hamamatsu). Representative H&Es matched the same FFPE block from which RNA was extracted. The assessment was made by a pathologist (RS) according to internationally established guidelines developed by the International Immuno-Oncology Biomarker Working Group (www.tilsinbreastcancer.org; ref. 32).

### Application of transcriptional signatures

We selected 4 transcriptional signatures related to DNA damage and repair pathways and 25 related to different aspects of immune biology which had been identified as being of interest in TNBC (Supplementary Table S1). All signatures were applied using the total RNA-seq gene expression data which passed quality control.

The CIN70 signature was applied as published (20). Gene expression data were log_2_ transformed, mean centred and the score calculated as the mean expression of the gene set made up of 70 genes. PARPi7 was applied as published including normalisation against mean expression of the normalization genes. In brief, the 7 signature genes were normalised against the geometric mean of the 7 normalization genes, log_2_ transformed and median centred before applying the formula using the weights and boundaries as published (22). The score was kept as a continuous score rather than dichotomised to retain full information. The gene expression based classifier for *TP53* mutation status was applied using the centroids from the original publication (21) with samples assigned to the nearest centroid using Spearman correlation. RPS was applied as published (23), log_2_ transformed gene expression values were median centred before calculating the score as the sum of the 4 signature genes multiplied by −1.

ConsensusTME individual cell type and average enrichment scores were calculated using the R code provided at https://github.com/cansysbio/ConsensusTME (33). DDIR signature formula was applied to the fpkm values using the weight and bias for each gene as originally published (34). IGG_cluster (35), B-cell T-cell cooperation (36), TFH signature (37), CD8 cluster (38), and T cells CD4 memory activated (39) are each calculated as the median expression of all genes (following log_2_ transformation) in the relevant signature. *PD-L1, CTLA4, PD1, LAG3, HAVCR2*, and *ENTPD1* are taken as the log2-transformed value for each individual gene.

For each of CD8 TRM, CD8 TRM mitotic, CD8 TEM, CD8 GD, CD4 TRM, CD4 CXCL13, CD4 FOXP3, CD4 IL7R, CD4 RGCC and monocytes, the lists of genes from the original publication (40) was filtered to genes with logfold change >1 and FDR *P* < 0.01 for the relevant cell type and the top 50 genes (based on logfold change) were selected. Examination of heatmaps of these gene sets applied to the TNT data indicated that for each cell type genes were not consistently expressed within patients so the scores were calculated as the first principal components for each cell type. The exception to this is CD8 TRM mitotic signature when genes were fairly homogeneously expressed within patients so the median gene expression was taken. For CAF_S1, all genes reported as upregulated specifically in the CAF_S1 subset (41) were selected in TNT and the first principal component calculated for the signature score.

Immune cell type abundance and diversity were deconvoluted using MiXCR (42).

### Statistical analysis

The association of signatures with HRD-associated characteristics was assessed using Wilcoxon rank sum. Associations of signatures with BRCAness were assessed using Kruskal–Wallis tests with *post hoc* pairwise comparisons via Dunn test. Categorical biomarkers were assessed using Fisher exact test and associations between continuous signatures were assessed using Spearman correlation.

The association of signatures with objective response rate (ORR) and PFS were assessed using logistic regression and restricted mean survival methods. To enable comparison across signatures, scores for each continuous biomarker were transformed to *Z*-scores prior to analysis of association with clinical outcomes.

To identify clusters based on expression of immune and DDR related signatures, conditional inference forest clustering (43) was performed integrating *BRCA1/2* mutation status and *BRCA1* methylation status, transcriptional signatures and TILs. Conditional inference forest clustering was applied using the R lumbRjacks package available at https://github.com/cancerbioinformatics/lumbRjacks. This was performed using a consensus clustering approach with 100 repetitions sampling 80% of the features in each iteration. The appropriate number of clusters was determined through examination of the consensus matrices and change in the area under the cumulative distribution function (CDF) curve.

To further characterize the novel clusters, we applied 693 previously defined gene expression “modules” (44) corresponding to different aspects of breast cancer biology. These are previously identified/defined sets of genes that are coexpressed (i.e., homogenously up- or downregulated) derived from different aspects of breast cancer biology. The signatures were collected from previous publications and gene set enrichment analysis as described in ref. 35 and 45. Module scores were calculated as the median expression of each gene within the module. We then performed hierarchical clustering of these modules supervised by the novel cluster assignment to visualize the expression of signatures related to other biological processes and visually compare this between the clusters. We also applied PAM50, Baylor (46), and TNBC subtypes (47) to assess how the novel clusters fit with existing subtypes.

Confidence intervals and *P* values were calculated as two-sided. Given the exploratory nature of these analyses, no adjustment has been made for multiple testing. Analyses were carried out in RStudio v.1.1.456 and Stata v.16.1.

This substudy was covered under the terms of the original consent and ethics approval. The TNT trial was approved by the East London and The City Main Research Ethics Committee and conducted according to the principals of Good Clinical Practice (GCP). Patients provided written informed consent.

### Assessing changes in an independent database

To assess signature score changes in an independent data set we used 2 publicly available RNA-seq datasets GEO databases GSE147322 (45) and GSE110590 (48). These datasets were filtered to only include patients whose primary tumor was triple negative and the second dataset was further subset to those where the primary tumour sample was taken pretreatment. Paired primary and metastatic samples were available for 11 patients, 5 of which had multiple metastatic samples profiled.

CIN70, RPS, and PARPi7 were applied as described above to these datasets. For each signature a hierarchical model was applied with the signature as the output, a random effect for patient (to account for some patients having multiple metastatic samples) and a fixed effect for timepoint (primary vs. recurrence) to test whether scores differ by timepoint.

### Data availability

Gene expression data have been deposited in the European Genome-phenome Archive (ID: EGAS00001007398). The datasets that support the findings of this study are subject to third party restrictions due to contractual agreements, and therefore, the clinical data is not publicly available. The datasets will be made available upon reasonable request. Data access requests are subject to approval, and should be addressed to M. Cheang (e-mail address: Maggie.Cheang@icr.ac.uk), A. Tutt (e-mail address: Andrew.Tutt@icr.ac.uk), and the TNT trial account (email address: tnt-icrctsu@icr.ac.uk).

## Results

### Patient characteristics

RNA sequencing data were available for primary tumor samples from 186 patients in the TNT trial (Fig. 1). Patient demographics in the patients evaluable in this biomarker substudy were generally comparable with the overall TNT population (*n* = 376); however, those within the evaluable cohort were more likely to have higher grade tumors, vascular invasion, and liver and lung metastases affecting the parenchyma. They were more likely to be within 5 years of initial diagnosis (Table 1). The majority of patients were *BRCA1/2* wild-type, and 24.2% displayed a germline or somatic *BRCA1* mutation or methylation (Table 1). *BRCA2* mutations are underrepresented in this genomic analysis subset (5 patients).

**Table 1.** Patient characteristics.

		RNA sequencing available							
		Carboplatin		Docetaxel		Total		Overall TNT population (total)	
		*n*	*%*	*n*	*%*	*n*	*%*	*n*	*%*
*BRCA1/2* status	*BRCA1* mutated	12	12.9	10	10.8	22	11.8	25	6.7
	*BRCA2* mutated	2	2.2	3	3.2	5	2.7	5	1.3
	*BRCA1* methylated	10	10.8	13	14.0	23	12.4	26	6.9
	*BRCA1/2* wildtype	56	60.2	54	58.1	110	59.1	124	33.0
	Uncertain	13	14.0	13	14.0	26	14.0	196	52.1
Age group	<40	9	9.7	11	11.8	20	10.8	35	9.3
	40–49	21	22.6	19	20.4	40	21.5	86	22.9
	50–59	34	36.6	37	39.8	71	38.2	130	34.6
	60+	29	31.2	26	28.0	55	29.6	125	33.2
Stage of disease	Metastatic	87	93.5	81	87.1	168	90.3	339	90.2
	Recurrent, inoperable, locally advanced	6	6.5	12	12.9	18	9.7	37	9.8
Performance status	0–1	84	90.3	86	92.5	170	91.4	350	93.1
	2	9	9.7	7	7.5	16	8.6	26	6.9
Prior taxane chemo	Yes	36	38.7	36	38.7	72	38.7	126	33.5
	No	57	61.3	57	61.3	114	61.3	250	66.5
Prior anthracycline for metastatic/locally advanced disease	Yes	4	4.3	7	7.5	11	5.9	36	9.6
	No	89	95.7	85	91.4	174	93.5	338	89.9
	Unknown	0	0.0	1	1.1	1	0.5	2	0.5
Liver/lung metastases affecting parenchyma	Yes	60	64.5	48	51.6	108	58.1	198	52.7
	No	33	35.5	45	48.4	78	41.9	178	47.3
Time from initial diagnosis to trial entry	0–1 from diag	9	9.7	16	17.2	25	13.4	68	18.1
	1–3 years from diag	55	59.1	49	52.7	104	55.9	189	50.3
	3–5 years from diag	24	25.8	19	20.4	43	23.1	74	19.7
	>5 years from diag	5	5.4	8	8.6	13	7.0	41	10.9
	Unknown	0	0.0	1	1.1	1	0.5	4	1.1
Nodal status	N-	42	45.2	28	30.1	70	37.6	146	38.8
	1–3N+	28	30.1	30	32.3	58	31.2	104	27.7
	≥4N+	22	23.7	31	33.3	53	28.5	81	21.5
	Unknown	1	1.1	4	4.3	5	2.7	45	12.0
Tumor grade	1	0	0.0	1	1.1	1	0.5	2	0.5
	2	10	10.8	12	12.9	22	11.8	57	15.2
	3	83	89.2	79	84.9	162	87.1	301	80.1
	Not known	0	0.0	1	1.1	1	0.5	16	4.3
Tumor size	<2 cm	19	20.4	17	18.3	36	19.4	82	21.8
	2–5 cm	57	61.3	64	68.8	121	65.1	208	55.3
	>5 cm	16	17.2	9	9.7	25	13.4	43	11.4
	Missing	1	1.1	3	3.2	4	2.2	43	11.4
Histologic subtype	Ductal/NST	88	94.6	88	94.6	176	94.6	337	89.6
	Lobular	1	1.1	1	1.1	2	1.1	9	2.4
	Other	4	4.3	4	4.3	8	4.3	18	4.8
	Missing	0	0.0	0	0.0	0	0.0	12	3.2

### Association between transcriptional DDR features, immune profile, and *BRCA*ness

**Figure 2.**
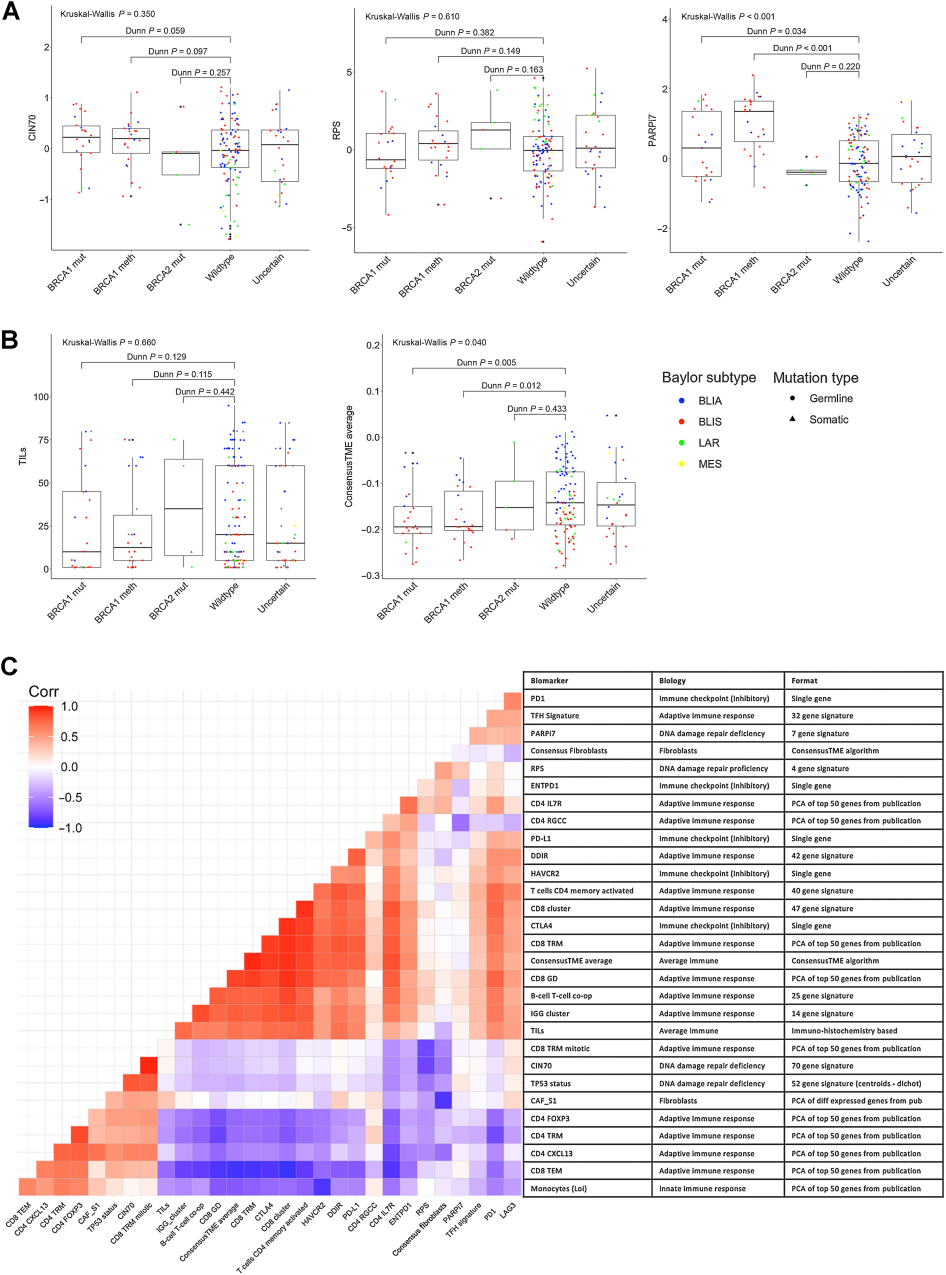
Overview of the characterization of DNA damage/repair and immune features in TNT samples and their association with each other. **A,** Distribution of DNA damage repair features by *BRCA1/2* status and Baylor subtype. **B,** Distribution of average immune infiltration features by *BRCA1/2* status and Baylor subtype. **C,** Correlation matrix of all signatures of interest.

We first explored whether expression of the 4 transcriptional DDR markers (Supplementary Table S1) differed according to *BRCA*ness assessed via both genetic and epigenetic mechanisms of loss of *BRCA1* or *BRCA2* function (*BRCA1* mutated/*BRCA1* methylated/*BRCA2* mutated/wild type) or HRD status (high/low as per Myriad's HRD signature) in this highly selected population of patients who developed advanced/metastatic disease. Neither CIN70 (20), transcriptional-based *TP53* signature (21) nor the RPS signature (23) were significantly associated with markers of *BRCA*ness or HRD (Fig. 2A; Supplementary Fig. S1A and S1B). PARPi7 scores (22) were higher in *BRCA1* mutated, or methylated tumours compared with *BRCA1/2* wild-type tumours (*P* < 0.001; Fig. 2A) and in HRD-high compared with low tumors (*P* < 0.001; Supplementary Fig. S1C), this remained true in *BRCA1/2* wild-type patients.

We also sought to test the association of features with number of telomeric allelic imbalances (NtAI), allelic imbalanced CNA (AiCNA), and high-level amplifications (HLAMP), three genomic scars previously shown to be associated with carboplatin response (17, 18).

Consistent with the lack of association with markers of *BRCA*ness, overall CIN70, transcriptional-based *TP53* and RPS were not associated with the genomic scars; however, RPS scores were higher in the 2nd tertile of AiCNA (Supplementary Fig. S1A and S1B). Higher PARPi7 scores were observed in the 2nd and 3rd tertiles of AiCNA compared with the first (test for trend *P* = 0.029; Supplementary Fig. S1C) and the low HLAMP group compared with high/none, although this was not statistically significant (*P* = 0.079; Supplementary Fig. S1C).

Next, we asked whether immune infiltration at the PT was associated with biomarkers of HR deficiency. While TILs assessed using H&E-stained sections were not associated with *BRCA1/2* germline and somatic mutation or methylation status (*P* = 0.660; Fig. 2B), higher TILs were observed in HRD-low compared with HRD-high tumours (*P* = 0.047; Supplementary Fig. S1D). Gene expression–based immune infiltration, assessed by ConsensusTME (33) average score, was lower in *BRCA1* mutated and *BRCA1* methylated tumours compared with *BRCA1/2* wild-type (*P* = 0.040; Fig. 2B) but did not differ according to HRD status (*P* = 0.720; Supplementary Fig. S1E).

We further explored the ConsensusTME individual cell type estimates to determine whether a particular cell type was driving the association with *BRCA1/2* status. Excluding fibroblasts, high positive correlation was observed between all cell types (Supplementary Fig. S1F) and a similar trend of association with *BRCA1/2* status was observed. Given the high correlation between different immune cell types by ConsensusTME, we used the average immune score and fibroblasts only for further analyses and explored additional, more specific, transcriptional based signatures of immune biology (Supplementary Table S1). Results using the individual cell types from Consensus TME were consistent with the average score (data not shown).

We explored the correlation between transcriptional markers of the DDR to better understand the breadth of information covered. CIN70 and RPS had a strong negative correlation, while CIN70 and transcriptional based *TP53* signature were positively correlated (Fig. 2C). PARPi7 was not correlated with the other transcriptional DDR markers (Fig. 2C). Two distinct groups of immune signatures were identified. The first group including *PD-L1*, CD8 cluster, and IGG cluster, was highly positively correlated with TILs, while CD4 FOXP3, CD4 TRM, CD4 CXCL13, and CD8 TEM were negatively correlated with the other group. Immune signatures were generally not correlated with DDR features (Fig. 2C).

### DNA damage and repair pathway or immune signatures as predictive biomarkers for response or PFS

Next, we asked whether any of the signatures were prognostic or predictive of treatment specific outcomes.

**Figure 3.**
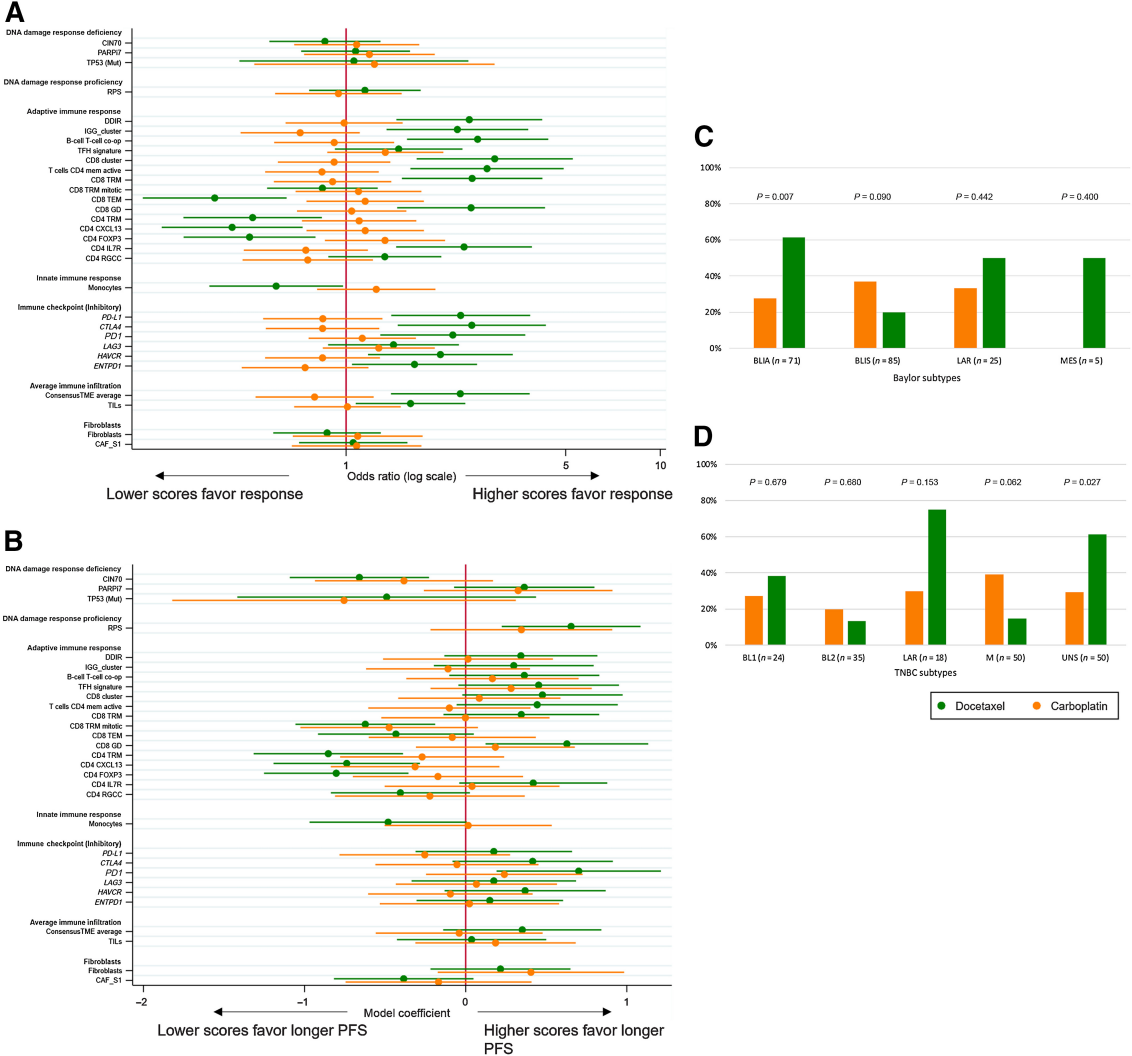
Association of biomarkers of interest with clinical outcomes by treatment group. **A,** Association of biomarkers with objective response. Odds ratios for each biomarker are presented from univariable logistic regression models. **B,** Association of biomarkers with PFS. Model coefficients of each biomarker are presented from linear regression of restricted mean PFS. Ninety-five percent confidence intervals are shown. Response rates are also presented by Baylor subtypes (**C**) and TNBC subtypes (**D**).

The transcriptional DDR signatures were not associated with differential response to either treatment (Fig. 3A; Supplementary Table S2).

High immune infiltration measured by TILs or gene expression–based signatures and gene expression-based immune checkpoint markers were predictive of response to docetaxel but not carboplatin (Fig. 3A; Supplementary Table S2). In particular, adaptive immune response markers were predictive of response to docetaxel; those positively correlated with overall immune infiltration were associated with an increased response with the opposite being true for signatures negatively correlated with immune infiltration (Fig. 3A; Supplementary Table S2). High monocyte scores (negatively correlated with immune infiltration) were associated with reduced response to docetaxel. No associations were observed between fibroblast scores and response for either treatment.

For analyses of the relationship between signatures and PFS, while nonsignificant, the direction of effects in the docetaxel group was consistent with those observed for objective response (Fig. 3B; Supplementary Table S2).

We explored the association between the Baylor subtypes (46) and TNBC subtypes (47) with clinical outcomes. Consistent with the TIME-related signatures, BLIA-classified TNBC had a preferential response to docetaxel. BLIS-classified TNBC had a superior response to carboplatin driven by a poor docetaxel response (Fig. 3C). No differences were observed for LAR or MES, although these subgroups were smaller. In the TNBC subtypes, tumors classified as UNS had a preferential response to docetaxel over carboplatin. There was a numerically higher response to docetaxel in LAR, while M classified tumours had a numerically higher response rate to carboplatin (Fig. 3D).

Nine patients entered the trial as germline *BRCA1/2* carriers who did not have TNBC, the conclusions were unaffected with these cases excluded from analysis.

### Understanding predictive effects in the context of prior chemotherapy

Given we are assessing transcriptional features in archival PT, but assessing response in the advanced disease setting, we hypothesized that the lack of association between biomarkers of aberrant DDR and increased response to carboplatin may be explained by changes in the DDR function in the tumor over the course of disease and under the selective pressure applied to micrometastatic disease clones by DNA-damaging adjuvant systemic chemotherapy. This may make the DDR status of the treatment naïve archival PT less relevant to the prediction of response in this advanced disease treatment setting.

**Figure 4.**
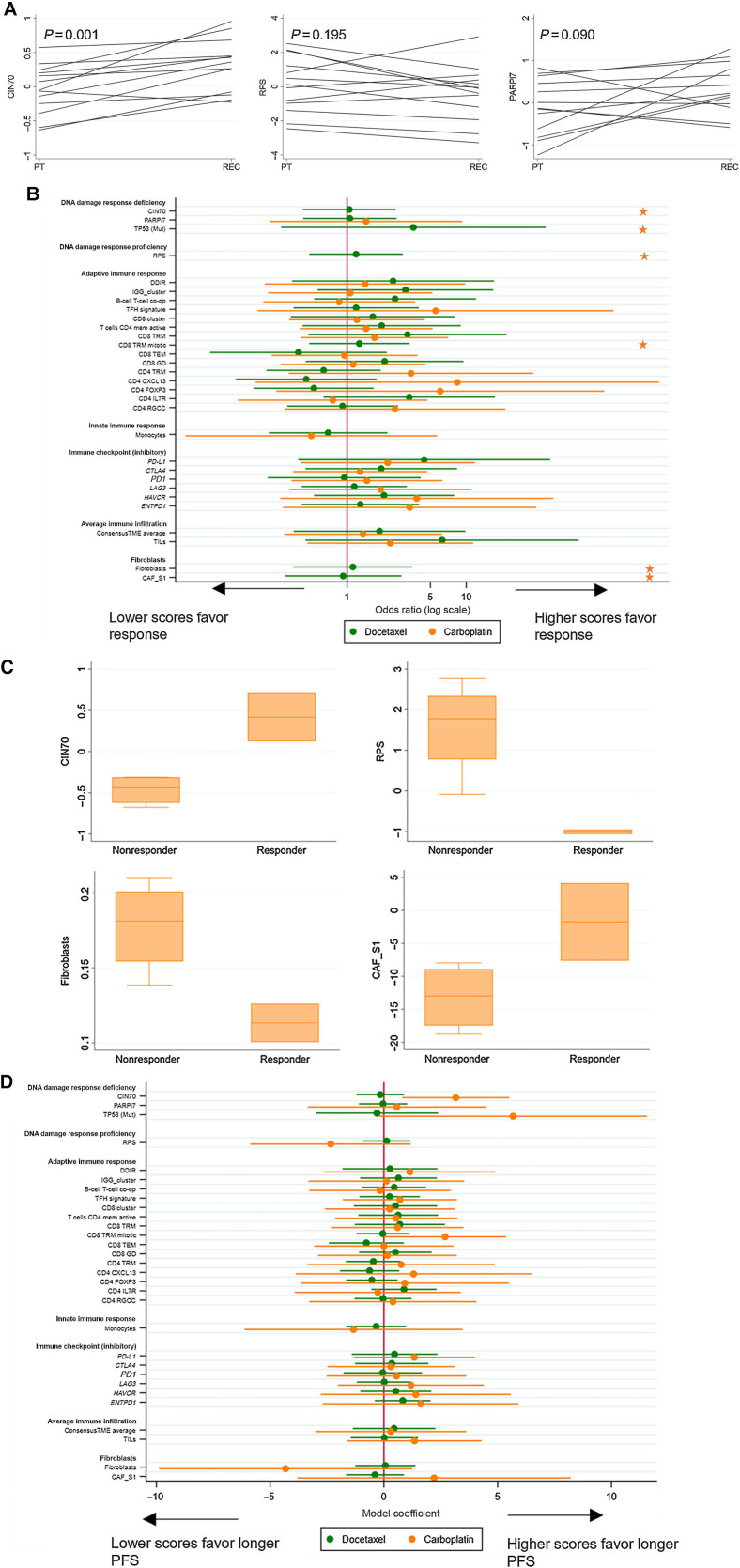
Association of biomarkers of interest with clinical outcomes by treatment group in chemotherapy-naïve patients. **A,** Changes in transcriptional DDR features between primary and recurrent disease. *P* values presented from *t* tests comparing primary and recurrent scores in paired samples. PT, primary tumor; REC, recurrent tumor. **B,** Association of biomarkers with objective response in chemotherapy-naïve patients. Odds ratios for each biomarker are presented from univariable logistic regression models. Stars indicate that an odds ratio could not be estimated due to colinearity (i.e., a cutoff could perfectly separate responders from nonresponders). **C,** Biomarker by response in chemotherapy-naïve, carboplatin-treated patients for biomarkers where a model could not be fitted due to a perfect cutoff between responders and nonresponders. **D,** Association of biomarkers with PFS in chemotherapy-naïve patients. Model coefficients of each biomarker are presented from linear regression of restricted mean PFS. Ninety-five percent confidence intervals are shown.

Using the 13 paired primary recurrent samples, we explored how the transcriptional DDR signatures changed from primary diagnosis to recurrence. All these patients received prior chemotherapy between primary diagnosis and trial entry. CIN70 was significantly higher in the recurrence than PT (mean change = 0.36; *P* = 0.001; Supplementary Table S3; Fig. 4A). Four of 6 *TP53* wild-type classified tumors were classified as mutant at recurrence, while all *TP53*-mutant classified samples retained the mutant classification. The RPS signature measure tended to decrease, although this did not reach statistical significance (mean change = 0.51; *P* = 0.195; Supplementary Table S3; Fig. 4A). Although there was not a consistent change in the PARPi7 signature, large changes were observed for some individuals (mean change = 0.44; *P* = 0.090; Supplementary Table S3; Fig. 4A). Changes in immune signatures were less consistent, although some demonstrated significant changes from primary to recurrent disease (Supplementary Table S3).

We further sought to validate the DDR signature changes in an independent dataset. For this, we used paired treatment-naïve PT with posttreatment metastatic samples from patients with TNBC from two publicly available RNA-seq datasets, GEO databases GSE147322 (45) and GSE110590 (see Supplementary Methods; ref. 48). This combined dataset demonstrated similar changes in transcriptional DDR signatures (Supplementary Fig. S2).

Given transcriptional DDR features do not appear to be preserved from primary to recurrent disease, and this may be related to the selective pressure induced by the use of chemotherapy in the early disease setting, we reassessed the association of these signatures with clinical outcomes separately by whether patients had received chemotherapy prior to entry in our trial.

Analysis in the prior chemotherapy treated cohort was generally consistent with results from the overall population (Supplementary Table S2).

In the smaller chemotherapy-naïve cohort (*n* = 21), CIN70, RPS, *TP53* mutation signature and fibroblast markers could separate responders and nonresponders treated with carboplatin (Fig. 4B and C; Supplementary Table S2) with no effect observed on docetaxel response. Of interest, the effects of fibroblasts and CAF_S1 signatures worked in opposite directions, suggesting the signatures identify different functions of fibroblasts. High CIN70 and *TP53*-mutant classified tumors were associated with numerically longer PFS (β coefficient for linear regression of restricted mean PFS = 3.16; 95% CI = 0.81–5.51; *P* = 0.020 and β = 5.68; 95% CI = −0.21–11.56; *P* = 0.055, respectively), while there was a trend to shorter PFS with high RPS and fibroblasts, although not statistically significant (β = −2.34; 95% CI = −5.86–1.18; *P* = 0.139 and β = −4.32; 95% CI = −9.88–1.24; *P* = 0.097, respectively). Significant biomarker treatment interactions were found for CIN70, *TP53*, and fibroblasts; patients with tumors expressing high DDR deficiency and high fibroblast signatures had longer PFS with carboplatin than docetaxel (Supplementary Table S2). In contrast, the PARPi7 signature and immune features were not predictive of response or PFS for either treatment group in this chemotherapy-naïve setting (Fig. 4B and D; Supplementary Table S2). Further analysis within *BRCA1/2*-defined subgroups would lead to insufficiently robust analyses and so, misleading conclusions have been avoided.

### Integration of multimodal data to identify novel biological subgroups

We sought to explore whether we could improve predictions by combining features to identify DDR and TIME biology defined subgroups. We performed unsupervised conditional inference clustering including all transcriptional DDR and immune features which were identified as having a significant association with response or PFS for either treatment group in any setting in univariable analyses (Supplementary Table S1). We also included TILs, *BRCA1/2* mutation and *BRCA1* methylation status as presented in the primary analysis report of the TNT Trial (1). Six clusters were identified (Supplementary Fig. S3), representing subgroups defined by TIME and DDR expression.

Cluster 1 (*N* = 63) and cluster 2 (*N* = 61) are predominantly defined by expression of the two sets of TIME-related markers. Cluster 1 includes tumors high for TILs and associated markers, cluster 2 includes tumors with low TILs but high expression of CD4 CXCL13/CD8 TEM signatures. Cluster 2 also had higher expression of CIN70 and higher rates of *TP53* mutated classified tumors compared with cluster 1. Cluster 3 (*N* = 20) is defined by high fibroblasts and high RPS with few *BRCA1/2* mutations, low CIN70, no *TP53* mutations, and low TILs. Cluster 5 (*N* = 21) is defined by high CIN70 with enrichment for *BRCA1* mutations and methylation and *TP53* mutations. These tumors also have relatively high TILs despite high expression of CD4 CXCL13/CD8 TRM mitotic signatures. Cluster 4 only contains 3 tumors, which have generally low/average expression of all markers and cluster 6 (*N* = 9) is comprised of all remaining tumors.

**Figure 5.**
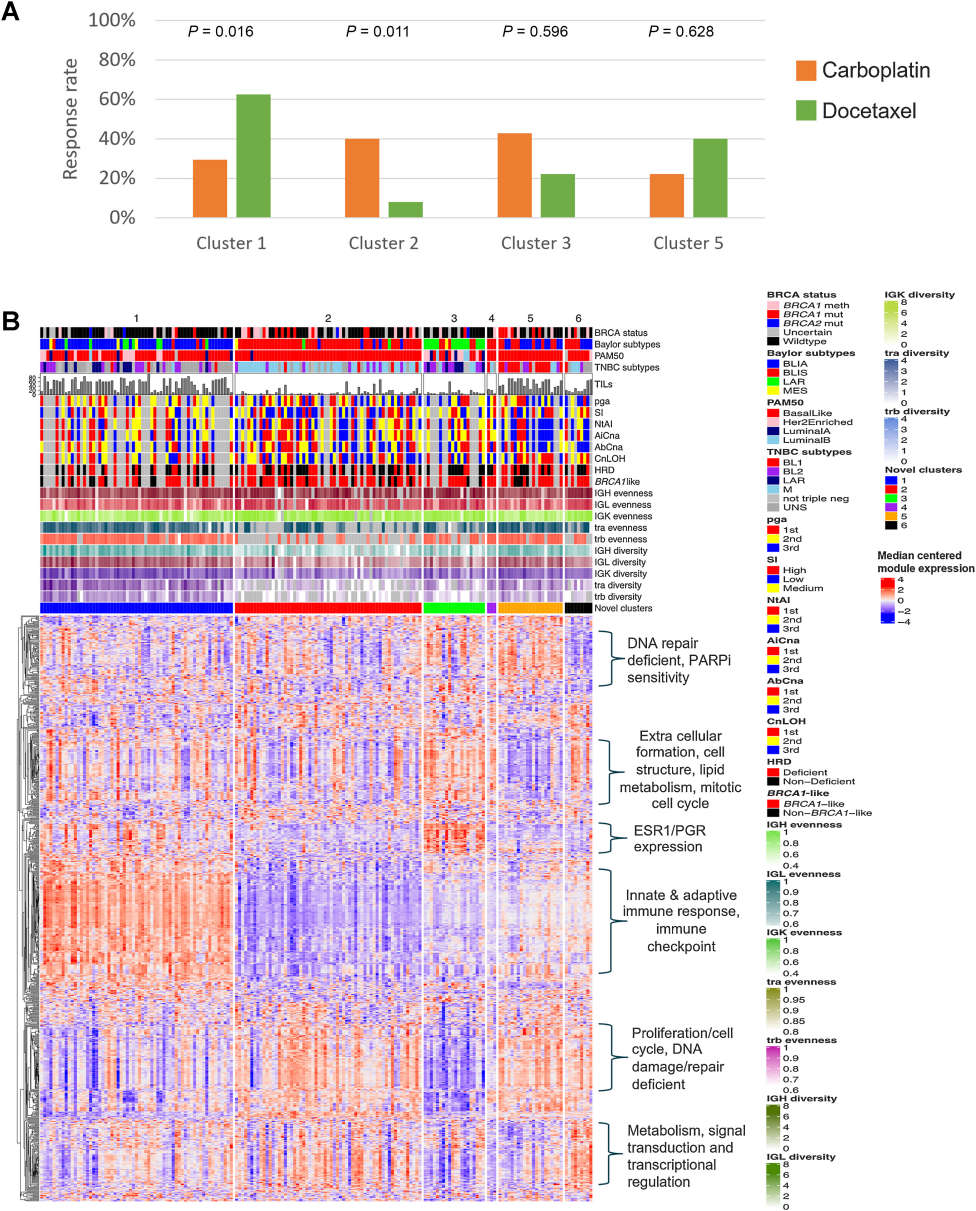
Results of a novel clustering. **A,** Response rates by novel clusters and treatment groups. *P* values are presented from Fisher exact tests. Clusters 4 and 6 are not shown due to small numbers. **B,** Heatmap showing biological features of each novel cluster. PGA, percentage of genome altered; SI, Shannon diversity index; NtAI, number of telomeric allelic imbalances; AiCna, allelic imbalanced CNA; AbCna, allelic balanced CNA; CnLOH, copy number neutral loss of heterozygosity.

In the prior chemotherapy exposed cohort, cluster 1 is associated with improved response rates with docetaxel over carboplatin (62.5% vs. 29.4%; *P* = 0.016; Fig. 5A) while cluster 2 showed the opposite (8.0% vs. 40.0%; *P* = 0.011; Fig. 5A). No significant differences are observed within the other clusters, but numbers are too small to draw firm conclusions. Analysis in the chemotherapy-naïve cohort is not presented, this would be inappropriate due to the small number of patients.

To characterize these clusters more comprehensively, we performed hierarchical clustering of a wider list of breast cancer–related module scores (Supplementary Methods) and genomic features supervised by cluster assignment (Fig. 5B). The heatmap was visually examined to identify the enrichment of modules related to other biological processes within and between each cluster. We also examined clinical characteristics and existing subtype classifications by cluster.

Cluster 1 tumors are predominantly basal-like immune activated, with high B-cell/T-cell diversity. No other pathways beyond immune biology appeared to be enriched in this cluster but patients were less likely to have visceral metastases (57% vs. 83% across other clusters) and more likely to have had high nodal involvement compared with some clusters (31% N4+ vs. 20% across clusters 2, 4, 5, and 6).

Cluster 2 tumors are primarily basal-like immune suppressed and mesenchymal (TNBC subtypes). Many of these tumours display high expression of proliferation and DDR pathway modules compared with cluster 1 and low expression of ESR1/PGR.

Cluster 3 has a high rate of HRD-nondeficident tumors as may be expected from high RPS scores and limited *BRCA1/2* deficiencies. They are predominantly nonbasal and mainly of the LAR subtype. These tumors display high expression of ESR1/PGR and markers of extracellular formation, cell structure, lipid metabolism, and mitotic cell cycle and low expression of proliferation. Cluster 3 also has high rates of nodal involvement (63% N4+), were lower grade at diagnosis (45% G1–2 vs. 9% across all other clusters), and includes older patients (65% aged 45+ vs. 25% across all other clusters).

Cluster 5 (*N* = 21) displays a high expression of proliferation markers. Tumors are largely basal-like and have high levels of NtAI and AiCNA. Tumors in cluster 5 display low expression of markers of extracellular formation, cell structure, lipid metabolism, and mitotic cell cycle as well as low expression of ESR1/PGR markers. We carried out a detailed pathology review of these cases (Supplementary Fig. S4) due to the contradictory TILs and TIME-related signature expressions. This confirmed high TIL levels and a high tumor–stromal area ratio. Morphologically, these cases displayed high-grade features including necrosis, high mitotic activity, high levels of atypia, and a solid growth pattern.

Cluster 6 (*N* = 9) is a mix of BLIA and BLIS tumors with high expression of modules related to metabolism, signal transduction and transcriptional regulation, and high expression of proliferation/cell cycle and low ESR1/PGR expression. Cluster 4 is too small to determine additional characteristics.

## Discussion

Overall, our study showed that transcriptional signatures related to immune pathways assessed in the PT can predict a selective docetaxel response compared with carboplatin in the advanced disease setting. However, transcriptional markers of DNA damage response did not predict sensitivity to either treatment in the overall population randomized in the trial.

It is important to acknowledge that a limitation of our study is that the TNT Trial bioresource is archival nontreatment-exposed material at the point of initial diagnosis in which we assessed all genomic and pathologic features and related these to response to randomized treatment in the often prior treatment exposed advanced/metastatic setting. The median time from diagnosis to trial entry was 2.1 years (IQR: 1.6–3.4 years), during which subclinical metastatic disease was exposed to the selective pressure of adjuvant chemotherapy or first-line anthracycline-based palliative chemotherapy in most patients. While reversion of germline mutations in *BRCA1/2* may have occurred (19), as can other forms of restoration of HR (49), germline mutation driven loss of HR appears to be preserved over the course of disease. This is evidenced by the significant interaction with a carboplatin-specific effect in this group (1). In contrast, it appears that wider DDR deficiency and transcriptional markers of this may be more plastic and reversible.

This is supported by our analyses of available paired primary and recurrent samples in TNT. Changes observed in DDR pathway related signatures suggest that metastatic tumours may have different transcriptional profiles to the primary diagnosis. Prior chemotherapy in the adjuvant setting may selectively target and eliminate DDR-deficient cells. However, resistant cells may remain and develop metastases, which were subjected to randomized treatment. Collection of metastatic tissue was not regularly done at the time of the study, and the small number of recurrent tumor samples is a limitation. By exploring two independent datasets, our theory that transcriptional markers of DDR deficiency in primary tumors may not accurately reflect the biology in the metastatic setting was confirmed. In support of this, we found in the chemotherapy-naïve cohort, CIN70, RPS, and *TP53* gene signatures were associated with clinical outcomes in patients treated with carboplatin. The small number of chemotherapy-naïve patients available for analysis is a constraint, and as a result, these findings should be considered hypothesis generating, and further studies in larger cohorts are warranted.

In contrast to the results of I-SPY2, which found that PARPi7 was predictive of pathologic complete response to the veliparib–carboplatin combination (50), the PARPi7 signature was not predictive of carboplatin response in either cohort. The I-SPY2 study, however, differs in several ways: (i) it was conducted in the early disease setting and included patients with hormone receptor positive disease; (ii) a combination of the PARP inhibitor veliparib with carboplatin compared with single-agent carboplatin in TNT was used; (iii) PARPi7 was developed from data in cell line models specifically aimed to predict response to PARP inhibitors. Despite evidence from the BRIGHTNESS trial that response to combination therapy is driven by the carboplatin component (5), it is likely that components of the PARPi7 signature may be specific to PARP inhibitor–driven effects leading to a lack of predictive performance for response to single-agent carboplatin treatment. In addition, the predictiveness of PARPi7 was not validated in BRIGHTNESS (51), and the authors hypothesize that the signature may not translate well from fresh frozen to FFPE (51) as used in TNT.

Given the lack of association between CIN70 and RPS with *BRCA1/2* and HRD status, despite higher RPS in intermediate AiCNA, it is possible that these signatures are not tracking DDR deficiencies but other biology. Many genes included in CIN70 are related to the mitotic cell cycle, so it has been postulated that CIN70 tracks proliferation (20). Within this cohort, CIN70 is highly correlated with the proliferation cluster (ρ = 0.96; ref. 35), and RPS is negatively correlated with this (ρ = −0.80) supporting this hypothesis.

We found that transcriptional signatures related to immune pathways and BLIA subtype were predictive of response to docetaxel in the metastatic setting. This is consistent with results from the early disease setting; the I-SPY2 trial showed immune signatures had broad predictive abilities across the majority of treatment arms, all of which included paclitaxel (51). Further to this, transcriptional signatures are perhaps stronger predictors than histopathologic based assessment of TILs as evidenced by the slightly more modest effect observed for TILs. This may be because TILs simply quantifies the presence of infiltrating lymphocytes, while transcriptional signatures convey the molecular characteristics in more detail, for example, to classify as T cells or B cells. While we explored immune related signatures covering different aspects of immune biology, the high correlation between signatures in this cohort hindered the identification of individual driving factors. We identified two distinct sets of immune features, despite other studies showing an association between CD4 CXCL13 and T-cell infiltration (52), our analyses showed that gene expression–based markers of CD4 CXCL13, CD8 TEM, CD4 TRM, and CD4 FOXP3 negatively correlated with TILs. One potential explanation considers the timing of sampling, with the timing of the T-cell–mediated immune response. As immune suppression increases (evidenced by increase CD4 FOXP3), antitumor TILs are suppressed. Over time, these effector TILs depart or die off due to prolonged immunosuppression, leaving only long-lived memory phenotypes (evidenced by TEM and TRM gene sets). Thus, we believe that increased expression of these genes sets, following prolonged immunosuppression, negatively correlates with TILs. This is confirmed in our study, where those cases with high/low TILs and low/high CXL13 had high/low levels of CD8 TRM, respectively. Conversely, if the sample contains TILs and less of these phenotypes, more of the immune system retains capacity to proliferate and, thus, clonally expand levels of TIL increase. CD4 CXCL13 cells also have a high indication of PD-1 expression (53), and therefore may represent an immunosuppressive phenotype in this cohort. Future work may be needed to explore the correlation of these cells with high-order immune-related structures such as TLS. Further immunohistopathologic, including multiplex immunofluorescence, analyses on the FFPE PT and metastatic material would be required to better dissect the spatially differential immune biology and drivers of response.

Immune infiltration has not been shown to predict survival in the advanced setting without the addition of immune checkpoint inhibitors. In Impassion130 and Keynote335, TILs and PD-L1 expression were predictive of longer PFS with atezolizumab/pembrolizumab but not standard chemotherapy (29, 54). Our results do not contradict this as the response did not translate to PFS benefit. One potential explanation is heterogeneous response within the tumor; death of chemotherapy-sensitive cells may lead to overall tumor shrinkage while resistant cells remain and develop metastases or progress. A better understanding and characterization of tumor heterogeneity in this highly selective population of TNBC patients could shed further light. Because RNA sequencing was carried out on bulk tumours, intratumor heterogeneity could not be assessed, and future spatial analysis including spatial transcriptomic analyses of both primary and recurrent disease would explore immune and epithelial heterogeneity and drivers of treatment resistance.

In contrast to the prediction of docetaxel response, we did not observe any association between immune markers and response to carboplatin. This is consistent with the results of several studies which show that immune scores (TILs or gene expression-based signatures) are predictive of chemotherapy response but this did not differ by carboplatin use in TNBC (26, 44, 55, 56). Each of these studies tested the addition of carboplatin to standard chemotherapy rather than as a single agent, limiting the ability to detect chemotherapy drug response–specific associations. Biomarkers of specific immune infiltrations may be predictive of differential responses to particular chemotherapies based on mechanism of action, induced cellular response and mode of cell death which differs between taxanes and platinum agents (57). For example, it is hypothesised that taxane effectiveness may not be solely due to microtubular inhibition in tumour cells but that they can also reduce T-regulatory cells leading to increased immune responses (31, 57).

Machine learning approaches utilising multimodal data identified the presence of 6 novel clusters amongst this cohort. Of particular interest is cluster 5 whose PT showed expression markers of DDR deficiency, coupled with moderate expression levels of immune cell markers, however high level of TILs (Fig. 5). Further pathologic assessment confirmed their high TIL contents and high-grade features, but also their solid architecture combined with basal-like breast cancer characteristics, confirming the gene expression findings. This combined analysis suggests that both gene expression signatures and evaluation of morphology, through a simple hematoxylin-eosin–stained slide, can extend the interpretation of the samples.

Cluster 2 demonstrated preferential response to carboplatin compared to cluster 1 driven by poor docetaxel response in cluster 2, likely due to the low expression of TILs despite high expression of CD4 CXCL13 and CD8 TEM signatures. Unfortunately, beyond the immune-defined clusters, sample sizes were too small to draw any firm conclusions regarding the sensitivity of these subgroups to either treatment. We clustered based on specific markers of TIME and DDR pathways, but an alternative approach would be to use the wider list of modules with a filter for those demonstrating a significant treatment interaction. As an exploratory exercise we applied this approach; it produced good overlap with our existing clusters; however, our original clusters 2 and 5 were combined into a single cluster (Supplementary Fig. S5) likely due to the increased number of immune features dominating the clustering. In the new clusters, the differential treatment effect previously observed in cluster 2 was diluted by inclusion of cluster 5 samples. This, and the lack of additional biological insights provided from the wider list of modules, supports our original approach of using the reduced list of well-characterized features which better dissected certain subgroups.

Our clusters showed some overlap with existing subtypes and demonstrated similar molecular features to those identified in I-SPY2, which also developed response predictive phenotypes based on DNA repair deficiency and immune biology. The study showed differential treatment responses between these groups for standard chemotherapy with/without carboplatin and veliparib or pembrolizumab in the early disease setting (51). Due to the similar characteristics of the clusterships developed by different approaches, reaching a consensus on TNBC subgroups could be key. An area of outstanding need which should be prioritised is to develop clinical studies to test novel agents for patients with TNBC with low immune infiltration and apparently DDR-proficient tumors, which show low response rates across current treatments in both our study and I-SPY2 (51). These patients will also likely be ineligible for pembrolizumab, which is now available as first-line metastatic treatment for patients with PD-L1–positive tumours. Although PD-L1 CPS was not available in this study, we assessed *PD-L1* gene expression. It is anticipated that PD-L1 CPS would largely overlap with the other immune signatures given the high correlation of *PD-L1* gene expression with TILs and other immune signatures.

To conclude, our study shows that biomarkers of high immune cell infiltration in primary tumor diagnostic material are associated with improved response to docetaxel but not carboplatin in the advanced setting. However, our results and those we have previously reported (1, 18) also highlight both the potential and complexity associated with the differential performance of biomarkers when applied to diagnostic material from primary cancer to predict response and treatment selection to mechanistically distinct therapies in the advanced disease setting. Future clinical trials in the metastatic setting exploring biomarkers of response should aim to use metastatic biopsies taken shortly before the investigational agent, in preference to archival primary tumor samples where possible to minimize this risk.

## Supplementary Material

Supplementary Figure S1 Additional associations between signatures. A. Distribution of CIN70 by HRD score and genomic scars (NtAI tertiles, AiCna tertiles and HLAMP). B Distribution of RPS by HRD score and genomic scars (NtAI tertiles, AiCna tertiles and HLAMP). C. Distribution of PARPi7 by HRD score and genomic scars (NtAI tertiles, AiCna tertiles and HLAMP). D. Distribution of TILs by HRD score, E. Distribution of ConcensusTME average score by HRD score F. ConsensusTME cell type estimates are highly correlated excluding fibroblasts.Correlation assessed using Spearman correlation. HRD<42 = HRD low; HRD≥42 = HRD high.

Supplementary Figure S2 Changes in A. CIN70, B. RPS and C. PARPi7 from treatment naïve primary tumours to post-treatment metastatic samples in an independent dataset. Multiple metastatic samples are included for some patients. 𝛃 coefficients and p-values presented for timepoint term from linear regression models with a random effect for patient to account for multiple samples from patients.

Supplementary Figure S3 Heatmap of signatures used for clustering by the resulting novel clusters.

Supplementary Figure S4 This is an example of DDR-deficient case with high TILs and low gene-expression measurements. These cases were confirmed to have high TIL content (black delineation) and are characterized by both high tumour area- stromal area ratio as well as a high tumour cell- stromal cell ratio. Moreover, all these cases were characterized by high grade features, such as necrosis (blue delineation), high mitotic activity (green arrow), and high levels of atypia (blue arrow), and all had a solid growth pattern, with no formation of glands.

Supplementary Figure S5 Heatmap showing clustering of all module scores filtered for a significant interaction with treatment. Our original clusters are shown against the new clusters at the top of the heatmap.

Supplementary Table S1 Supplementary table 1 - Biomarkers of interest

Supplementary Table S2 Supplementary table 2 - Model estimates for logistic regression models of objective response rate and linear regression models of restricted PFS by treatment group

Supplementary Table S3 Supplemetary table 3 - Change in signature scores from primary to recurrence

Supplementary Table S4 Supplementary Table 4 - Representativeness of Study Participants
