# Supplementary material for: Integrated Multimodal Analyses of DNA Damage Response and Immune Markers as Predictors of Response in Metastatic Triple-Negative Breast Cancer in the TNT Trial (NCT00532727)
Source: Clin Cancer Res. 2023 Aug 14;29(18):3691–705. doi: 10.1158/1078-0432.CCR-23-0370 (PMC10502473; doi:10.1158/1078-0432.CCR-23-0370)
Supplement: Supplementary Figure S1 — Additional associations between signatures. A. Distribution of CIN70 by HRD score and genomic scars (NtAI tertiles, AiCna tertiles and HLAMP). B Distribution of RPS by HRD score and genomic scars (NtAI tertiles, AiCna tertiles and HLAMP). C. Distribution of PARPi7 by HRD score and genomic scars (NtAI tertiles, AiCna tertiles and HLAMP). D. Distribution of TILs by HRD score, E. Distribution of ConcensusTME average score by HRD score F. ConsensusTME cell type estimates are highly correlated excluding fibroblasts.Correlation assessed using Spearman correlation. HRD<42 = HRD low; HRD≥42 = HRD high. [file ccr-23-0370_supplementary_figure_s1_suppfs1.pdf]

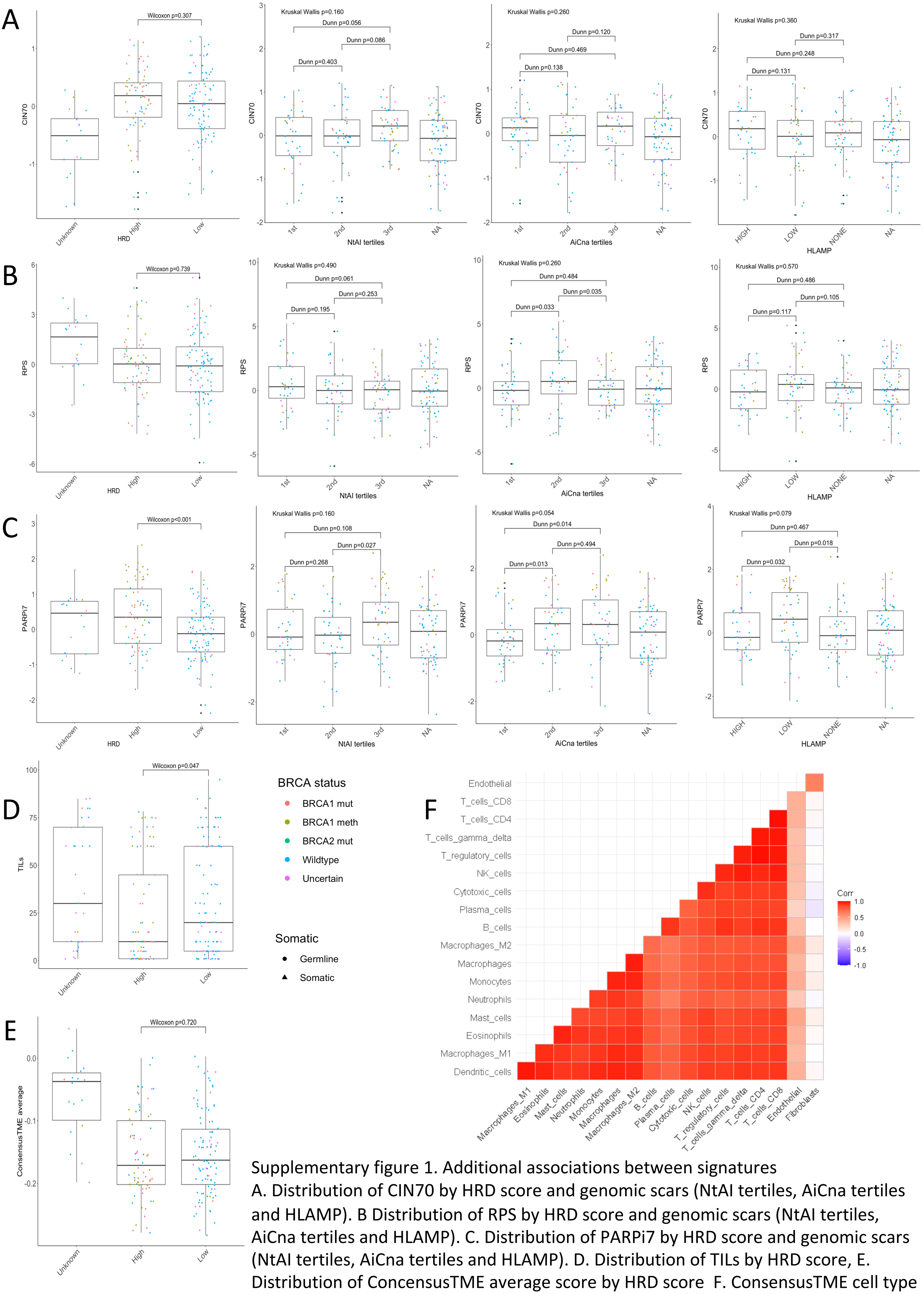

Supplementary figure 1. Additional associations between signatures
A. Distribution of CIN70 by HRD score and genomic scars (NtAI tertiles, AiCna tertiles and HLAMP). B Distribution of RPS by HRD score and genomic scars (NtAI tertiles, AiCna tertiles and HLAMP). C. Distribution of PARPi7 by HRD score and genomic scars (NtAI tertiles, AiCna tertiles and HLAMP). D. Distribution of TILs by HRD score, E. Distribution of ConcensusTME average score by HRD score F. ConsensusTME cell type estimates are highly correlated excluding fibroblasts.Correlation assessed using Spearman correlation. HRD<42 = HRD low; HRD≥42 = HRD high.
