# Supplementary material for: Integrated Multimodal Analyses of DNA Damage Response and Immune Markers as Predictors of Response in Metastatic Triple-Negative Breast Cancer in the TNT Trial (NCT00532727)
Source: Clin Cancer Res. 2023 Aug 14;29(18):3691–705. doi: 10.1158/1078-0432.CCR-23-0370 (PMC10502473; doi:10.1158/1078-0432.CCR-23-0370)
Supplement: Supplementary Figure S2 — Changes in A. CIN70, B. RPS and C. PARPi7 from treatment naïve primary tumours to post-treatment metastatic samples in an independent dataset. Multiple metastatic samples are included for some patients. 𝛃 coefficients and p-values presented for timepoint term from linear regression models with a random effect for patient to account for multiple samples from patients. [file ccr-23-0370_supplementary_figure_s2_suppfs2.pdf]

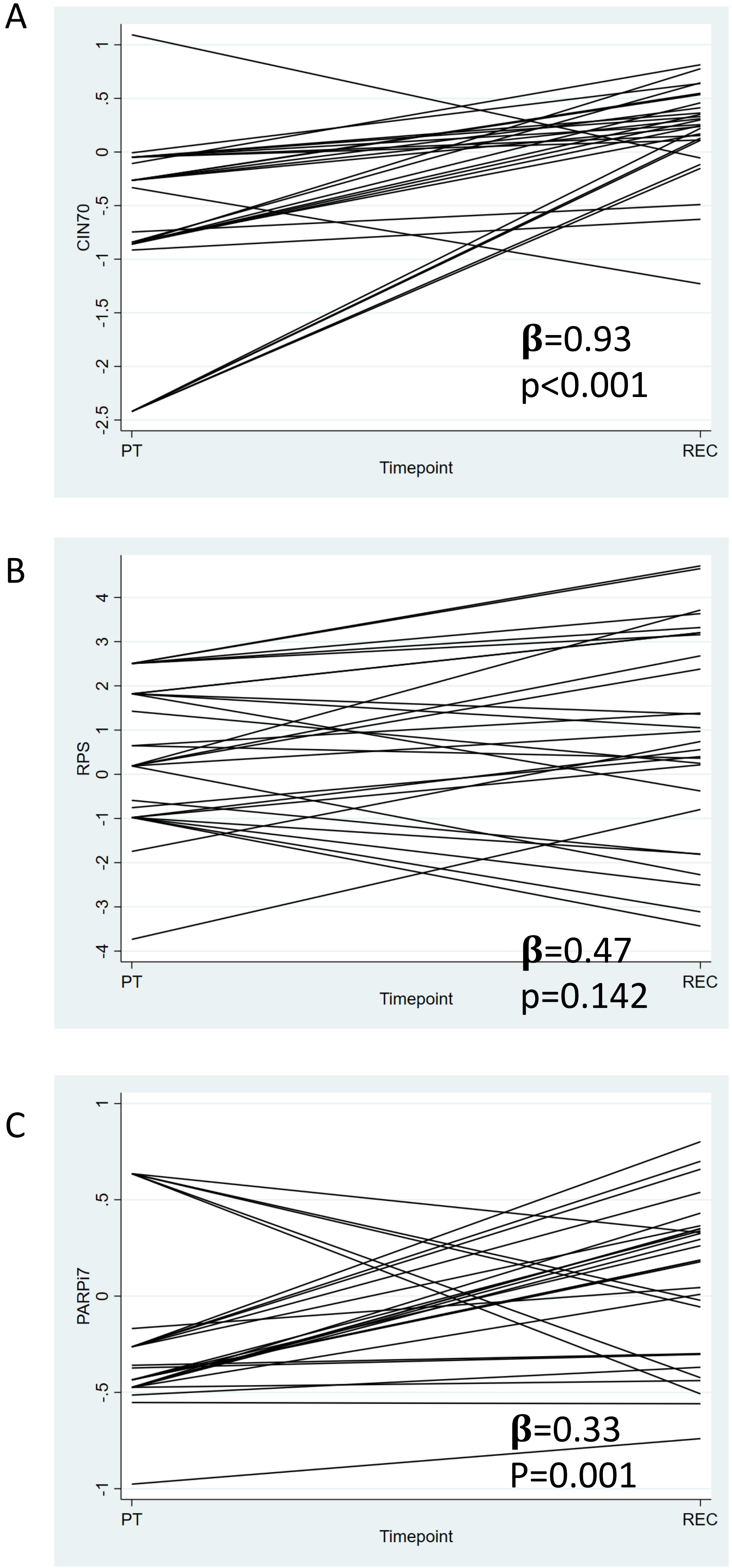

Supplementary figure 2.
Changes in A. CIN70, B. RPS and C. PARPi7 from treatment naïve primary tumours to post-treatment metastatic samples in an independent dataset.
Multiple metastatic samples are included for some patients.
β coefficients and p-values presented for timepoint term from linear regression models with a random effect for patient to account for multiple samples from patients.
