# Supplementary material for: Integrated Multimodal Analyses of DNA Damage Response and Immune Markers as Predictors of Response in Metastatic Triple-Negative Breast Cancer in the TNT Trial (NCT00532727)
Source: Clin Cancer Res. 2023 Aug 14;29(18):3691–705. doi: 10.1158/1078-0432.CCR-23-0370 (PMC10502473; doi:10.1158/1078-0432.CCR-23-0370)
Supplement: Supplementary Figure S3 — Heatmap of signatures used for clustering by the resulting novel clusters. [file ccr-23-0370_supplementary_figure_s3_suppfs3.pdf]

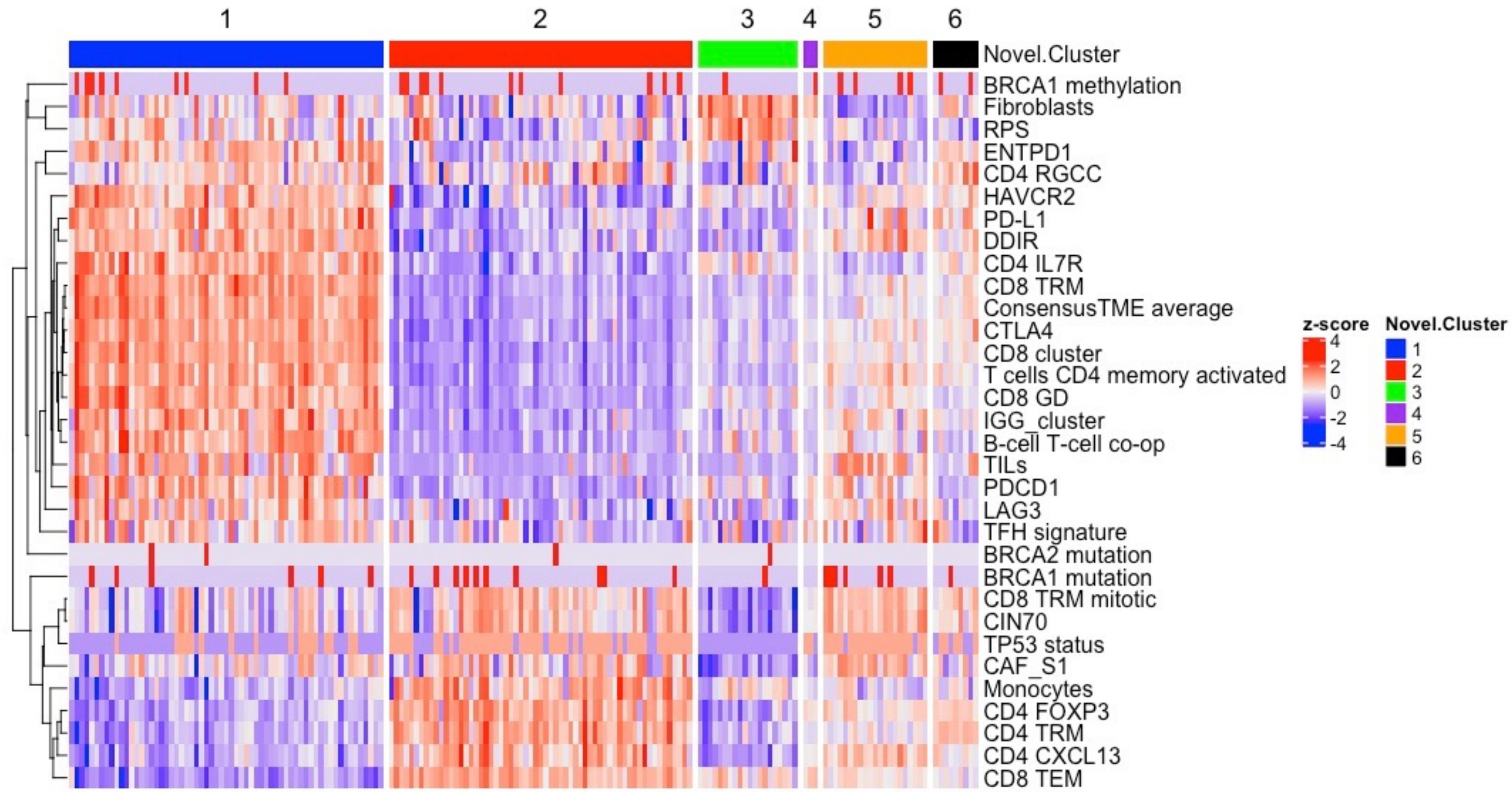

Supplementary figure 3. Heatmap of signatures used for clustering by the resulting novel clusters.
