# Supplementary material for: Integrated Multimodal Analyses of DNA Damage Response and Immune Markers as Predictors of Response in Metastatic Triple-Negative Breast Cancer in the TNT Trial (NCT00532727)
Source: Clin Cancer Res. 2023 Aug 14;29(18):3691–705. doi: 10.1158/1078-0432.CCR-23-0370 (PMC10502473; doi:10.1158/1078-0432.CCR-23-0370)
Supplement: Supplementary Figure S4 — This is an example of DDR-deficient case with high TILs and low gene-expression measurements. These cases were confirmed to have high TIL content (black delineation) and are characterized by both high tumour area- stromal area ratio as well as a high tumour cell- stromal cell ratio. Moreover, all these cases were characterized by high grade features, such as necrosis (blue delineation), high mitotic activity (green arrow), and high levels of atypia (blue arrow), and all had a solid growth pattern, with no formation of glands. [file ccr-23-0370_supplementary_figure_s4_suppfs4.pdf]

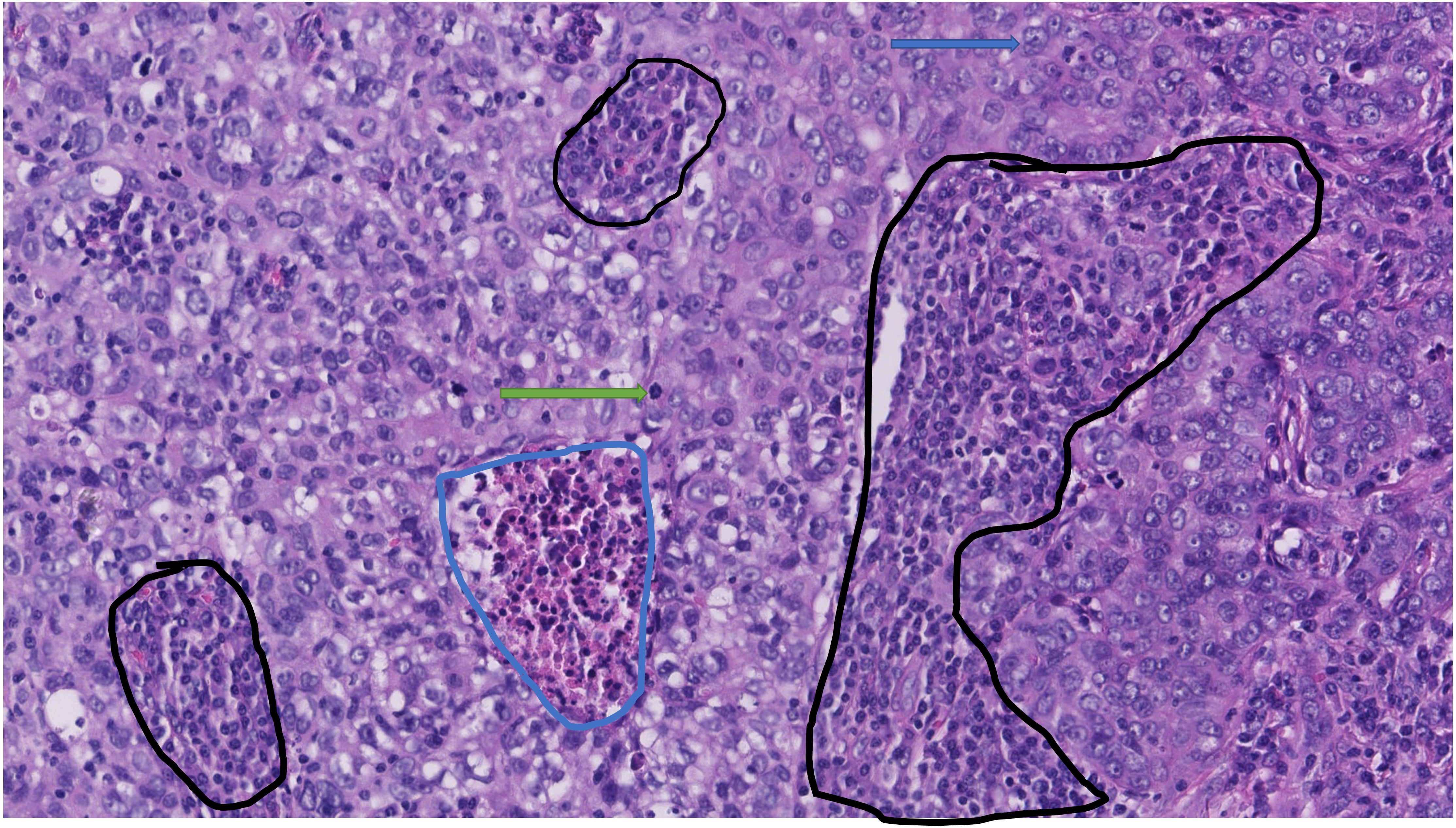

Supplementary figure 4. This is an example of DDR-deficient case with high TILs and low gene-expression measurements. These cases were confirmed to have high TIL content (black delineation) and are characterized by both high tumour area- stromal area ratio as well as a high tumour cell- stromal cell ratio. Moreover, all these cases were characterized by high grade features, such as necrosis (blue delineation), high mitotic activity (green arrow), and high levels of atypia (blue arrow), and all had a solid growth pattern, with no formation of glands.
