# Supplementary material for: Integrated Multimodal Analyses of DNA Damage Response and Immune Markers as Predictors of Response in Metastatic Triple-Negative Breast Cancer in the TNT Trial (NCT00532727)
Source: Clin Cancer Res. 2023 Aug 14;29(18):3691–705. doi: 10.1158/1078-0432.CCR-23-0370 (PMC10502473; doi:10.1158/1078-0432.CCR-23-0370)
Supplement: Supplementary Figure S5 — Heatmap showing clustering of all module scores filtered for a significant interaction with treatment. Our original clusters are shown against the new clusters at the top of the heatmap. [file ccr-23-0370_supplementary_figure_s5_suppfs5.pdf]

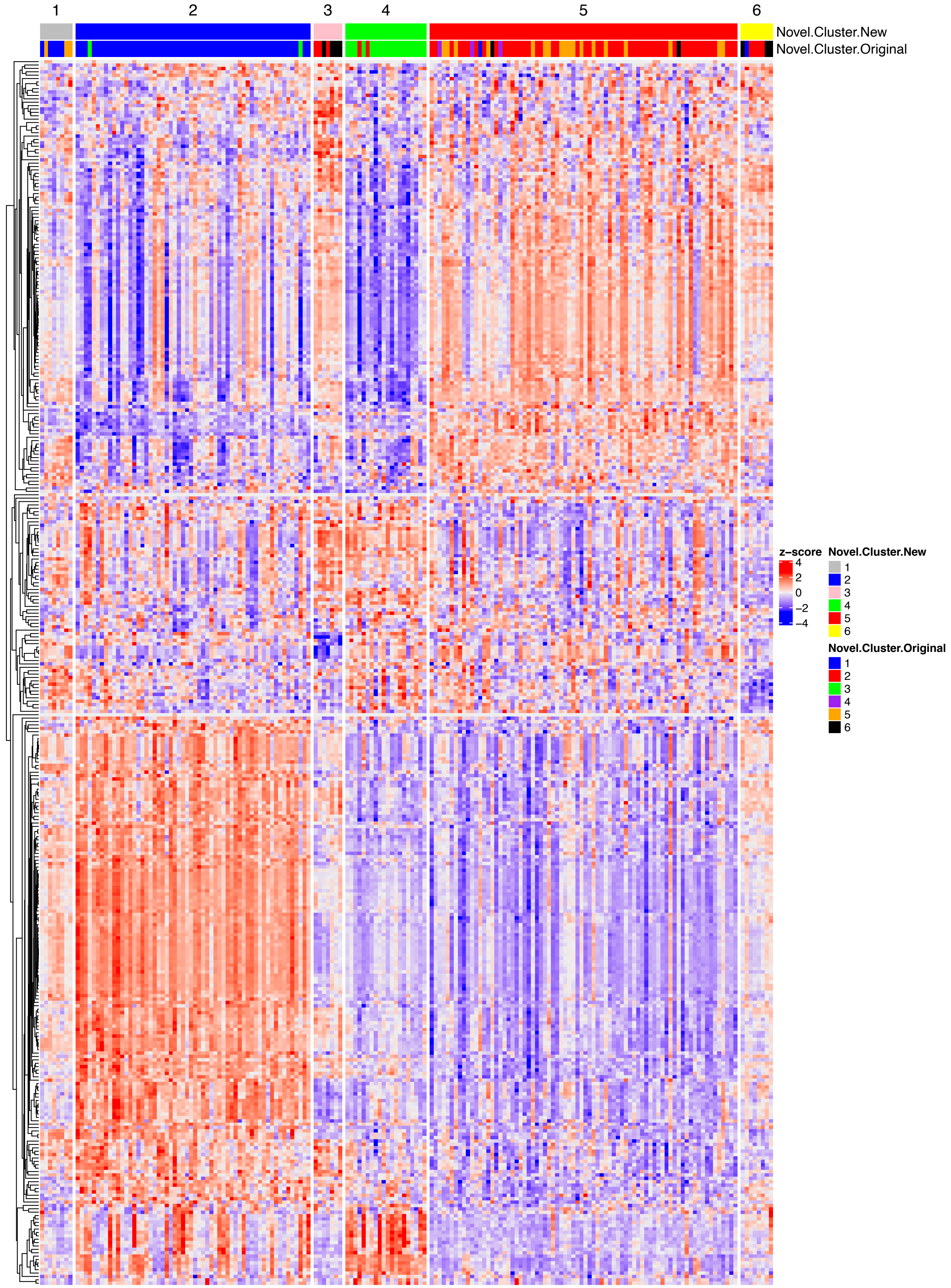

Supplementary figure 5. Heatmap showing clustering of all module scores filtered for a significant interaction with treatment. Our original clusters are shown against the new clusters at the top of the heatmap.
