# Supplementary material for: Integrated Multimodal Analyses of DNA Damage Response and Immune Markers as Predictors of Response in Metastatic Triple-Negative Breast Cancer in the TNT Trial (NCT00532727)
Source: Clin Cancer Res. 2023 Aug 14;29(18):3691–705. doi: 10.1158/1078-0432.CCR-23-0370 (PMC10502473; doi:10.1158/1078-0432.CCR-23-0370)
Supplement: Supplementary Table S1 — Supplementary table 1 - Biomarkers of interest [file ccr-23-0370_supplementary_table_s1_suppts1.docx]

**Supplementary table 1. Biomarkers of interest**

| **Category** | **Signature Name** | **Description** | **Hypothesis** | **Included in integrated analyses?** |
| --- | --- | --- | --- | --- |
| DNA damage response deficiency - Transcriptional | CIN70(1) | Continuous signature developed using functional aneuploidy as a surrogate for chromosomal instability. Genes were ranked by their correlation with functional aneuploidy and top 70 genes selected. Calculated as the average of these genes. Higher scores indicate greater instability. | Patients with higher scores will benefit from carboplatin over docetaxel. | Yes |
|  | PARPi7(2) | Continuous signature developed using logistic regression of genes reported to be involved in DNA repair or PARP inhibitor response to predict olaparib response in cell lines. Higher scores associated with greater probability of response. | Patients with higher scores will benefit from carboplatin over docetaxel. | No (not significant in univariable analysis is any setting) |
|  | TP53 status (Mutant vs. Wildtype)(3) | Centroid-based dichotomous signature developed using functional p53 in cell lines and *P53* status in tumour samples. Patients classified as TP53 mutant/wildtype. | Patients classified as TP53 mutant will benefit from carboplatin over docetaxel. | Yes |
|  | RPS(4) | Continuous signature score developed in cancer cell lines. Genes related to replication stress and the DSB repair pathway were correlated with resistance to topotecan as a surrogate for HR proficiency to reduce the gene list. Low RPS score is associated with high DDR deficiency. | Patients with lower scores will benefit from carboplatin over docetaxel. | Yes |
| DNA damage response deficiency - Genomic | BRCA1 mutation status | Germline/tumour BRCA1 mutation - classed as Mutant vs. wildtype | N/A - analysis previously reported(5). | Yes |
|  | BRCA1 methylation status | Tumour BRCA1 methylation - classed as Methylated vs. wildtype |  |  |
|  | BRCA2 mutation status | Germline/tumour BRCA2 mutation - classed as Mutant vs. wildtype |  |  |
| Adaptive immune response - Transcriptional | DDIR(6) | Developed as a DNA damage response deficiency classification based on presence of molecular processes associated with Fanconi anaemia. Subsequently noted that the signature is characterised by immune gene expression(7). | Patients with higher scores have higher immune infiltration and will have greater benefit from either drug compared to patients with low scores. | Yes |
|  | IGG_cluster(8) | Identified through unsupervised clustering of breast cancer samples, 14 genes associated with immunoglobulin. Shown to be associated with good prognosis in breast cancer. |  |  |
|  | B-cell T-cell co-op(9) | Developed through supervised clustering of ICC sensitive and resistant tumours from mouse models. A cluster of genes related to immune cell activity, B cell genes, IgGs, and T cell genes. High scores suggest high immune cell gene expression. |  |  |
|  | TFH signature(10) | Developed in colorectal cancer using publicly available data to compare transcriptional profile of different immune cells including T-follicular helper cells. Higher signature score suggests higher presence of TFH cells. Subsequently shown to predict survival in breast cancer. |  |  |
|  | CD8 cluster(11) | Developed using TCGA mRNAseq breast cancer data, unsupervised hierarchical clustering identified gene sets associated with specific immune cell types, including one for CD8+ T-cells. High signature scores suggest high presence of CD8+ T-cells. |  |  |
|  | T cells CD4 memory activated(12) | From Cibersort, immune cell deconvolution method using linear support vector regression to estimate relative expression of immune cell types. High expression suggests high expression of T cells memory activated relative to other immune cell types. |  |  |
|  | CD8 TRM(13) | Developed through single cell sequencing of TNBC tumour samples. Unsupervised clustering identified 10 unique clusters including one representing:  CD8 tissue resident memory T cells. | Patients with higher scores will have greater benefit from either drug compared to patients with low scores. | Yes |
|  | CD8 TRM mitotic(13) | Developed as above representing CD8 tissue resident memory T cells with mitotic features. |  |  |
|  | CD8 TEM(13) | Developed as above representing CD8 tissue T effector memory cells. |  |  |
|  | CD8 GD(13) | Developed as above representing CD8+ gamma delta cells. |  |  |
|  | CD4 TRM(13) | Developed as above representing CD4 tissue resident memory T cells. |  |  |
|  | CD4 CXCL13(13) | Developed as above representing CD4+CXCL13+ cells. |  |  |
|  | CD4 FOXP3(13) | Developed as above representing T-cells CD4+FOXP3+ |  |  |
|  | CD4 IL7R(13) | Developed as above representing CD4+ IL7R+ cells |  |  |
|  | CD4 RGCC(13) | Developed as above representing CD4+ RGCC+ cells. |  |  |
| Innate immune response - Transcriptional | Monocytes(13) | Developed as above representing monocytes. | Patients with higher scores will have greater benefit from either drug compared to patients with low scores. | Yes |
| Immune checkpoint (Inhibitory) - Transcriptional | PD-L1 | Single gene expression. | Patients with higher scores will have greater benefit from either drug compared to patients with low scores. | Yes |
|  | CTLA4 |  |  |  |
|  | PD1 |  |  |  |
|  | LAG3 |  |  |  |
|  | HAVCR2 |  |  |  |
|  | ENTPD1 |  |  |  |
| General/average immune - Transcriptional | ConsensusTME average(14) | Consensus gene sets created from existing deconvolution methods. The gene sets were then used to generate normalised enrichment scores for different immune cell types. This is the average across immune cell types. | Patients with higher scores will have greater benefit from either drug compared to patients with low scores. | Yes |
| General/average immune - H&E | TILs | Assessed as the percentage of stromal area occupied by tumour infiltrating lymphocytes. Assessed on H&E stained slides. | Patients with higher scores will have greater benefit from either drug compared to patients with low scores. | Yes |
| Fibroblasts - Transcriptional | Fibroblasts(14) | Consensus gene sets created from existing deconvolution methods. The gene sets were then used to generate normalised enrichment score for fibroblasts. | Patients with higher scores will have greater benefit from either drug compared to patients with low scores. | Yes |
|  | CAF_S1(15) | Four subpopulations of cancer associated fibroblasts were identified via flow cytometry including an immuno-suppressive subset CAF_S1. A list of genes upregulated in this subset of cells was identified. | Patients with higher scores will have greater benefit from either drug compared to patients with low scores. | Yes |

**References**

1. Carter SL, Eklund AC, Kohane IS, Harris LN, Szallasi Z. A signature of chromosomal instability inferred from gene expression profiles predicts clinical outcome in multiple human cancers. Nat Genet. 2006;38(9):1043-8.

2. Daemen A, Wolf DM, Korkola JE, Griffith OL, Frankum JR, Brough R, et al. Cross-platform pathway-based analysis identifies markers of response to the PARP inhibitor olaparib. Breast Cancer Res Treat. 2012;135(2):505-17.

3. Troester MA, Herschkowitz JI, Oh DS, He X, Hoadley KA, Barbier CS, et al. Gene expression patterns associated with p53 status in breast cancer. BMC Cancer. 2006;6:276.

4. Pitroda SP, Pashtan IM, Logan HL, Budke B, Darga TE, Weichselbaum RR, et al. DNA repair pathway gene expression score correlates with repair proficiency and tumor sensitivity to chemotherapy. Sci Transl Med. 2014;6(229):229ra42.

5. Tutt A, Tovey H, Cheang MCU, Kernaghan S, Kilburn L, Gazinska P, et al. Carboplatin in BRCA1/2-mutated and triple-negative breast cancer BRCAness subgroups: the TNT Trial. Nat Med. 2018;24(5):628-37.

6. Mulligan JM, Hill LA, Deharo S, Irwin G, Boyle D, Keating KE, et al. Identification and validation of an anthracycline/cyclophosphamide-based chemotherapy response assay in breast cancer. J Natl Cancer Inst. 2014;106(1):djt335.

7. Parkes EE, Walker SM, Taggart LE, McCabe N, Knight LA, Wilkinson R, et al. Activation of STING-Dependent Innate Immune Signaling By S-Phase-Specific DNA Damage in Breast Cancer. J Natl Cancer Inst. 2017;109(1).

8. Fan C, Prat A, Parker JS, Liu Y, Carey LA, Troester MA, et al. Building prognostic models for breast cancer patients using clinical variables and hundreds of gene expression signatures. BMC Med Genomics. 2011;4:3.

9. Hollern DP, Xu N, Thennavan A, Glodowski C, Garcia-Recio S, Mott KR, et al. B Cells and T Follicular Helper Cells Mediate Response to Checkpoint Inhibitors in High Mutation Burden Mouse Models of Breast Cancer. Cell. 2019;179(5):1191-206 e21.

10. Bindea G, Mlecnik B, Tosolini M, Kirilovsky A, Waldner M, Obenauf AC, et al. Spatiotemporal dynamics of intratumoral immune cells reveal the immune landscape in human cancer. Immunity. 2013;39(4):782-95.

11. Iglesia MD, Vincent BG, Parker JS, Hoadley KA, Carey LA, Perou CM, et al. Prognostic B-cell signatures using mRNA-seq in patients with subtype-specific breast and ovarian cancer. Clin Cancer Res. 2014;20(14):3818-29.

12. Newman AM, Liu CL, Green MR, Gentles AJ, Feng W, Xu Y, et al. Robust enumeration of cell subsets from tissue expression profiles. Nat Methods. 2015;12(5):453-7.

13. Savas P, Virassamy B, Ye C, Salim A, Mintoff CP, Caramia F, et al. Single-cell profiling of breast cancer T cells reveals a tissue-resident memory subset associated with improved prognosis. Nat Med. 2018;24(7):986-93.

14. Jimenez-Sanchez A, Cast O, Miller ML. Comprehensive Benchmarking and Integration of Tumor Microenvironment Cell Estimation Methods. Cancer Res. 2019;79(24):6238-46.

15. Costa A, Kieffer Y, Scholer-Dahirel A, Pelon F, Bourachot B, Cardon M, et al. Fibroblast Heterogeneity and Immunosuppressive Environment in Human Breast Cancer. Cancer Cell. 2018;33(3):463-79 e10.
