# Supplementary material for: Integrated Multimodal Analyses of DNA Damage Response and Immune Markers as Predictors of Response in Metastatic Triple-Negative Breast Cancer in the TNT Trial (NCT00532727)
Source: Clin Cancer Res. 2023 Aug 14;29(18):3691–705. doi: 10.1158/1078-0432.CCR-23-0370 (PMC10502473; doi:10.1158/1078-0432.CCR-23-0370)
Supplement: Supplementary Table S4 — Supplementary Table 4 - Representativeness of Study Participants [file ccr-23-0370_supplementary_table_s4_suppts4.docx]

| **Supplementary Table 4.** Representativeness of Study Participants | |
| --- | --- |
| Cancer type(s)/subtype(s)/stage(s)/condition | Triple negative breast cancer (TNBC) |
| Considerations related to: | |
| Sex | TNBC is rare in men. Overall, TNBC counts for approximately 15% of breast cancer diagnoses but only ~3% in men(1). |
| Age | TNBC is more common in younger women than other breast cancer subtypes, with a mean age at diagnosis of 53(2). |
| Race/ethnicity | TNBC rates tend to be higher in non-Hispanic black women compared to other ethnic groups(3). |
| Geography | In the UK, there are estimated to be >8000 new cases of TNBC each year. |
| Other considerations | TNBC tends to be more aggressive than other subtypes of breast cancer, with larger tumours and higher grade tumours at diagnosis. |
| Overall representativeness of this study | The age distribution within our study is as expected for patients with TNBC.  Only women were recruited to the study but TNBC is very rare in males. |

1. Leone JP, Leone J, Zwenger AO, Iturbe J, Vallejo CT, Leone BA. Prognostic significance of tumor subtypes in male breast cancer: a population-based study. Breast Cancer Res Treat. 2015;152(3):601-9.

2. Dent R, Trudeau M, Pritchard KI, Hanna WM, Kahn HK, Sawka CA, et al. Triple-negative breast cancer: clinical features and patterns of recurrence. Clin Cancer Res. 2007;13(15 Pt 1):4429-34.

3. Kohler BA, Sherman RL, Howlader N, Jemal A, Ryerson AB, Henry KA, et al. Annual Report to the Nation on the Status of Cancer, 1975-2011, Featuring Incidence of Breast Cancer Subtypes by Race/Ethnicity, Poverty, and State. J Natl Cancer Inst. 2015;107(6):djv048.
